# The efficacy and safety of intravenous tranexamic acid in anterior cruciate ligament reconstruction: A randomized controlled trial protocol

**DOI:** 10.1097/MD.0000000000021747

**Published:** 2020-08-21

**Authors:** Hongyao Xu, Pengcheng Xia, Xiangjie Zou, He Huang

**Affiliations:** Department of sports and joint, Nanjing Hospital Affiliated to Nanjing Medical University (Nanjing first hospital), Jiangsu, China.

**Keywords:** anterior cruciate ligament reconstruction, blood loss, complications, protocol

## Abstract

**Background:**

The safety and efficacy of intravenous tranexamic acid (TXA) in the anterior cruciate ligament (ACL) reconstruction remains controversial. There is an urgent need of studies that efficiently control for confounding, conduct comprehensive and consecutive observation of potential risks of the TXA administration, and investigate its clinical applicability. The purpose of this work is to assess the safety and efficacy of the intravenous TXA in decreasing perioperative blood loss in the patients undergoing ACL reconstruction.

**Methods:**

This randomized, controlled, prospective research was carried out between January 2017 and January 2018. All the patients and their family members signed the informed consent forms, and this current work was authorized via the ethics committee of Nanjing first hospital (registration No.: NJU1003586). A total of 100 patients were divided randomly into 2 group: the control group (n = 50) and study group (n = 50). The study group receives intravenous TXA administration [1 g] before skin incision. The control group receives equivalent normal saline. Primary outcome measures including blood loss, hemoglobin decline, transfusion rate, C-reactive protein, D-dimer value, fibrinogen, prothrombin time, activated partial thromboplastin time, thrombin time, international normalized ratio and erythrocyte sedimentation rate were recorded. The measures of secondary outcomes refer to the clinical data involving the range of motion and postoperative pain score. The pain score was quantified by utilizing the 10-cm scale of visual analog. The pain strength was in the range of 0–10, where 0 is totally no pain and 10 represents the most severe pain.

**Results:**

This experiment had strict inclusive criteria and exclusive criteria and a well- regulated intervention.

**Conclusion:**

Our results can bring a new perspective on the use of TXA after arthroscopically assisted ACL surgery.

**Trial registration:**

This study protocol was registered in Research Registry (researchregistry5798).

## 1 Introduction

The arthroscopically assisted anterior cruciate ligament (ACL) reconstruction has become a prevalent surgical procedure in recent years.^[1,2]^ The number of ACL reconstruction has increased with the development of technology and concept. It is reported that more than 175 thousand primary ACL reconstruction procedures are performed annually in the United States.^[3]^ Although the ACL reconstruction is regarded as a minimally invasive and relatively safe surgery, it still possesses potential complications.^[4,5]^ Perioperative blood loss may be associated with numerous adverse effects including increased susceptibility to infection, potential toxic effects to the cartilage, possible subsequent synovitis, and increased scar formation with delayed rehabilitation.^[6]^ Blood transfusion may cause some adverse reactions, such as infection, immune response, myocardial infarction, as well as increase the medical expenses of patients.^[7–9]^ Minimizing blood loss in knee arthroscopy is crucial for improving surgical outcomes. In order to solve these problems, tourniquets and antifibrinolytic drugs are commonly used to reduce the loss of blood volume in perioperative period.

Tranexamic acid (TXA), a lysine derivative with a structure similar to that of lysine, can be combined with lysine targets on the plasminogen and plasminogen to block the interaction between fibrin and heavy chain of plasmin, thus promoting the coagulation process and achieving the goal of controlling postoperative blood loss.^[10,11]^ In clinical practice, TXA is often injected intravenously or intraarticular to reduce perioperative blood loss in orthopedics surgery.^[12,13]^ In addition, some studies have shown that TXA can not only reduce blood loss, but also play a significant role in inflammatory response.^[14,15]^ The safety of intravenous TXA has been extensively studied in knee arthroplasty surgery for several years. Xiong et al^[16]^ reported that combined administration of intravenous and topical TXA did not increase the rates of deep venous thrombosis or pulmonary embolism. However, evidence of intravenous TXA in ACL reconstruction is lacking. The purpose of this work is to assess the safety and efficacy of the intravenous TXA in decreasing perioperative blood loss in the patients undergoing ACL reconstruction. We hypothesized that TXA acted as an effective hemostatic agents after ACL reconstruction without increasing the incidence of thrombosis events.

## 2 Materials and methods

### 2.1 Participants

100 patients prepared for ACL reconstruction in the Nanjing first hospital were enrolled in the study between January 2017 and January 2018. The inclusion criteria for this trial included that

(1) the clinical diagnosis was ACL rupture;
(2) there was no thrombus in both lower limbs before operation;
(3) hemoglobin (HB) and coagulation indexes were normal before operation.

Exclusion criteria included that

(1) the patient had coagulation dysfunction;
(2) the patient had a history of knee infection or severe deformation of the knee joint;
(3) the patient was allergic to TXA or drugs involved in surgery;
(4) preoperative color doppler ultrasound of both lower limbs indicated thrombus;
(5) the patient is not able to tolerate the operation due to poor physical function.

All the patients and their family members signed the informed consent forms, and this current work was authorized via the ethics committee of Nanjing first hospital (registration No.: NJU1003586). This trial was registered in research registry (researchregistry5798).

100 participants were divided randomly into 2 groups, namely, study group (n = 50) and control group (n = 50), respectively. A table of random numbers hidden in the 1:1 ratio was computer-formed via a nurse. The randomization was blind, it was conducted via nurses in sealed envelopes before operation. And at the same time, TXA was prepared by a senior anesthesiologist. None of the nurses and anesthesiologists were involved. And in this present trial, the patients, surgeons, researchers as well as statisticians were all blinded. Intervention group receives intravenous TXA administration [1 g] before skin incision. The control group receives equivalent normal saline.

### 2.2 Surgical methods

Preoperative tourniquet was routinely used in all patients and systolic blood pressure was kept at about 300 mm Hg. Each surgery was conducted by the same qualified surgeon in the same institution. Combined spinal anesthesia was performed.

All the patients were given the short-term antibiotic prophylactic treatment 30 minutes before the operation, with a single injection of 2 g of cefazolin. All the patients underwent single bundle autografts of hamstring muscle to reconstruct ACL. Standard double-anterior arthroscopic approach was utilized. The anterior tibial cruciate ligament was reconstructed via proximally fixing graft utilizing the Milagro Advance Interference Screws (DePuy Mitek) and Rigidfix ACL Cross Pin System (DePuy Mitek, Raynham, MA) for the fixation of tibial. Any ancillary surgery, if conducted, was registered (such as meniscus tear repair or cartilage changes). The operation time and tunnel diameter were both recorded.

The patients were routinely monitored by low-flow oxygen inhalation, electrocardiographic monitoring, blood oxygen saturation and blood pressure within 24 hours after the surgery. The area around incision was treated with intermittent cold compress for 48 hours. Twelve hours after surgery, the patients were subcutaneously given 0.4 mL/d of low molecular weight heparin sodium until 10 days after surgery. If the patient has the following conditions, blood transfusion was required. The blood transfusion indications were: the hemoglobin less than 70 g/L or the patient had such anemia symptoms as dizziness, palpitation and mental malaise although hemoglobin was 70 to 100 g/L.^[17]^

### 2.3 Observation indicators

Patients underwent blood routine examinations before and post-operation of the 1st, 3rd and 6th day. Primary outcome measures including blood loss, hemoglobin decline, transfusion rate, C-reactive protein, D-dimer value, fibrinogen, prothrombin time, activated partial thromboplastin time, thrombin time, international normalized ratio and erythrocyte sedimentation rate were recorded. The measures of secondary outcome refer to the clinical data involving the range of motion and postoperative pain score. The pain score was quantified by utilizing the 10-cm scale of visual analog.^[18]^ The pain strength was in the range of 0 to 10, where 0 is totally no pain and 10 represents the most severe pain.

### 2.4 Statistical analysis

The calculations of sample size are conducted utilizing the software of PASS 2011 (NCSS, LLC, Kaysville, UT). The requirements of sample size are detected on the main results of the decrease in the hemoglobin concentration, which is on the basis of our former data on same determinations. In order to determine the difference in 1.0 g/L of the major endpoint, 49 patients were required in each group at the alpha of 0.05 and a power of 0.90. All the needed analyses are implemented through utilizing SPSS for Windows Version 20.0. All the data are represented with proper characteristics as median, mean, percentage as well as standard deviation. Mann-Whitney *U* test or the independent samples t-test were used to analyze the inter group comparison. Chi-square detection was utilized to compare the categorical variables among the groups. The analysis of repeated measurement of the variance was applied to analyze the repeated data. A *P* < .05 was regarded the significant in statistics.

## 3 Results

The results will be shown in Tables 1 and 2.

**Table 1:**
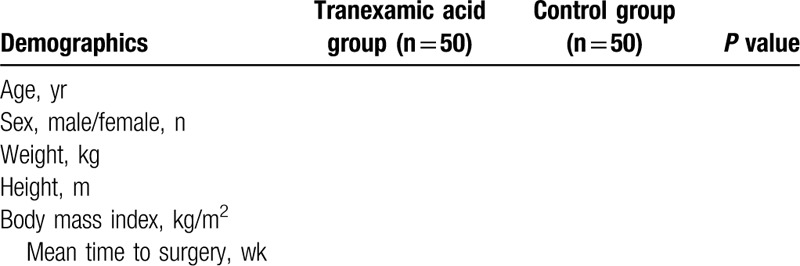
Patient characteristics.

**Table 2:**
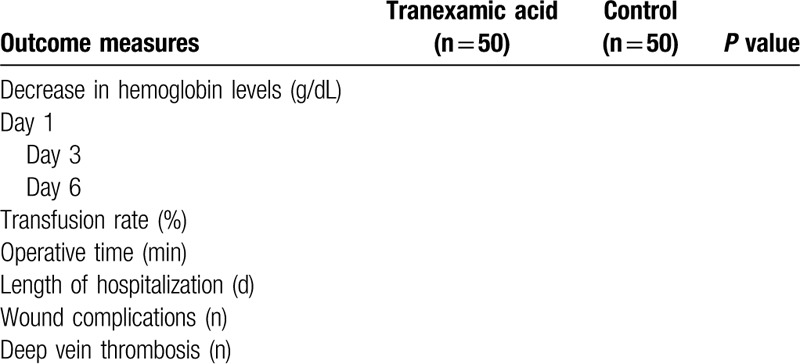
Study results.

## 4 Discussion

Arthroscopically assisted ACL reconstruction is a common and reproducible procedure. Different strategies have been established to decrease the complication rate in these patients, and hemarthrosis is 1 of these undesired complications. The application of TXA to decrease postoperative and surgical blood loss and subsequent blood transfusion requirements has been well described in a variety of orthopedic surgeries, but the researches have not identified any method for the arthroscopic operation. We conducted this randomized controlled trial to determine an effective TXA protocol for patients undergoing arthroscopic reconstruction of ACL. Hemarthrosis is toxic to the articular cartilage and enhances the susceptibility to the infections.^[19]^ Although postoperative hemarthrosis is usually associated with immediate morbidity, it can ultimately lead to poor results. Even if the follow-up period is sufficient to test the clinical results, such a short follow-up period cannot be used to evaluate early side effects after reconstruction of ACL. Future research with large sample size is still necessary.

## 5 Conclusion

Our results can bring a new perspective on the use of TXA after arthroscopically assisted ACL surgery.

## Author contributions

Pengcheng Xia planned the study design. He Huang reviewed the study protocol. Xiangjie Zou will recruit participants and collect data. Hongyao Xu wrote the manuscript. All of the authors have read, commented on, and contributed to the submitted manuscript.
